# Comparing the Use of Uterine Artery Embolization to Gonadotropin-Releasing Hormone Agonists in Shrinking Fibroid Size: A Pilot Study in Kazakhstan

**DOI:** 10.5195/cajgh.2015.232

**Published:** 2015-09-17

**Authors:** Balkenzhe Imankulova, Alibek Mereke, Nazira Kamzaeva, Talshyn Ukybassova

**Affiliations:** 1 Department of Obstetrics and Gynecology, National Research Center for Maternal and Child Health, National Medical Holding, Astana, Kazakhstan;; 2 Department of Epidemiology, Graduate School of Public Health, University of Pittsburgh, Pittsburgh, PA

**Keywords:** fibroids, Kazakhstan, GnRHa, uterine artery embolization

## Abstract

**Introduction:**

Uterine fibroids are the most common benign tumor in women in Kazakhstan. In the past two decades, endoscopic surgery has played an important role in the development of gynecologic surgery, particularly in the treatment of uterine fibroids. The goal of this paper is to evaluate whether uterine artery embolization (UAE) or gonadotropin-releasing hormone agonists (GnRHa) prior to myomectomy was more effective in decreasing fibroid size and improving surgical outcomes in a pilot study of women in Kazakhstan.

**Methods:**

This pilot investigation included 24 patients separated into 2 groups: medication group (pre-treatment with GnRHa – 13 patients) and embolization group (pre-treatment with UAE – 11 patients). All patients had uterine fibroids, 3–10 cm in diameter, and were treated with myomectomy at the National Research Center for Maternal and Child Health, Astana, Kazakhstan. All patient data were obtained by a retrospective medical records review. Descriptive statistics were utilized to characterize participant demographics data. Independent t-tests were used to analyze continuous variables, and Chi-square and Fisher’s exact tests were used where appropriate for count data.

**Results:**

The group treated with GnRHa had an operating time of 40±10 minutes longer than the group treated with UAE, due to the peri-operative difficulties encountered by surgeons in detecting the layer between the myometrium and fibroid capsule. The group treated with UAE experienced better patient outcomes (less blood loss, less surgical time, and reduced use of anesthesia) and was a technically easier surgery due to visible differences in uterine layers.

**Conclusions:**

Despite the fact that both treatments (GnRHa and UAE) were effective for fibroid shrinking, embolization resulted in more optimal surgical time and improved patient outcomes. Results of this pilot study need to be confirmed in a randomized clinical trial, specifically focused on Kazakhstan and the Central Asian Region.

Approximately 20–30% of women worldwide over the age of 30 are diagnosed with uterine fibroids.^1^,^2^ Uterine fibroids are the most common benign tumor diagnosed in women in Kazakhstan; however, treatment of this problem remains a complex and difficult challenge. The true prevalence of fibroids is likely to be underestimated in Kazakhstan, as access to medical facilities, and medical care in general, varies across the country.

Symptomatic fibroids are associated with significant morbidity and are typically associated with prolonged and heavy periods, pelvic pain, and in some cases, reproductive problems.^3^ Surgical interventions, such as hysterectomy or laparotomic myomectomy, used to be the primary surgical interventions for uterine fibroids. Other methodologies have been emerging in the past several years, such as laparoscopic myomectomy and uterine artery embolization (UAE), as well as medicinal interventions to reduce the fibroid volume. Hysterectomy is a curative approach for fibroid management; however, patients of reproductive age are in need of interventions that preserve reproductive function. With access to proper laparoscopic equipment and availability of well trained surgeons, laparoscopic surgery is the treatment of choice for symptomatic fibroids. Progesterone and its receptors increase the proliferation of fibroid cells in the uterus; therefore, antiprogestins and progesterone receptor modulators are considered reasonable medicinal treatments.^2^

Gonadotropin-releasing hormone agonists (GnRHa) are commonly used before hysteroscopic myomectomy to make surgery easier and safer, but they are expensive, have potential side effects, and lack evidence based data to support this practice.^4^ Therapy using GnRHa appears to result in a decrease in estrogen and progesterone levels, which decrease the size of the fibroid While GnRHa do cause fibroid regression, they can only be used in the short term, as temporary measures in the perimenopausal women, or pre-operatively to reduce fibroid size before myomectomy.^5^ The disadvantages of using GnRHa reported in the literature are the rebound growth of the fibroids upon cessation of therapy and major side effects associated with their use.^5^ In a previously published systematic review of the literature on the use of pre-operative GnRH analogues for symptom relief, GnRHa may improve some outcomes, but there is insufficient evidence to support their routine use prior to hysteroscopic resection of submucous fibroids.^4^

While GnRHa are typically believed to be useful in fibroid reduction,^2^ our experience (unpublished data) suggests that fibroids exposed to agonists have deeper myometrium invasion that significantly impairs their enucleation during surgery. A previously published study suggested that pre-surgical treatment with embolization prior to myomectomy has the benefits of lowering intra-operative blood loss and increasing efficacy of conservative myomectomy.^6^ Additional benefits of UAE include reduction in the volume of fibroids and their vascularization, thus reducing surgical time. Furthermore, patients undergoing UAE appear to have shorter recovery times and fewer clinical symptoms, such as pain and fever due to resorption, as well as a decrease in uterine size.^6^,^7^

Despite the fact that multiple papers and reviews have been published on the efficacy of GnRHa and UAE on fibroid reduction, many unanswered questions still exist in this area. Also, no studies have been conducted in Kazakhstan or Central Asia to compare the use of GnRHa to UAE. Thus, our study fills a significant gap in the research by comparing the pre-treatment with uterine artery embolization (UAE) to gonadotropin releasing hormone agonists (GnRHa) prior to myomectomy in their effectiveness in improving surgical outcomes, specifically focusing on women in Kazakhstan.

## Methods

This is a retrospective review of 24 clinical cases treated at the National Research Center for Maternal and Child Health in Astana, Kazakhstan from 2013–2014. In 2012, this center became one of the first clinics in the Commonwealth of Independent States, which was accredited by the Joint Commission International. This study was limited to women of reproductive age with symptomatic fibroids undergoing laparoscopic myomectomy. Before myomectomy, all women were pre-treated for fibroid volume reduction.

These 24 cases were divided into two groups, medication group and embolization group, which were based on the pre-surgical treatment of the fibroids. The medication group included 13 women who underwent laparoscopic myomectomy after pre-treatment with GnRHa on an outpatient basis in domiciliary clinics. GnRHa was injected intramuscularly every 4 weeks for a period of 8 weeks (3 doses) prior to surgical intervention. Conservative myomectomy was carried out 4 weeks after the last injection. The embolization group included 11 patients who underwent laparoscopic myomectomy 6–12 months after UAE. UAE was carried out at the National Research Center for Maternal and Child Health on an inpatient basis. The National Research Center for Maternal and Child is a tertiary treatment facility specializing in gynecologic and obstetrical conditions. UAE procedures have been carried out at the center since 2008. Because this study was limited to the review of medical records, it was exempt from human subject IRB review. Prophylactic antibiotic therapy (cefazolin, 2000 mg intravenously, 30 minutes before the surgery) was administered to all patients, based on standard treatment protocol. Patients came for follow-up assessment 6 to 12 months after the surgery, where vaginal examinations and transvaginal uterine ultrasounds were conducted to assess fibroid symptoms and reduction.

### Data Analysis

Descriptive statistics were utilized to characterize participant demographics data. Independent t-tests were used to analyze continuous variables, and Chi-square and Fisher’s exact tests were used where appropriate for count data. All analyses were conducted in Microsoft Excel.

## Results

**Figure 1.**
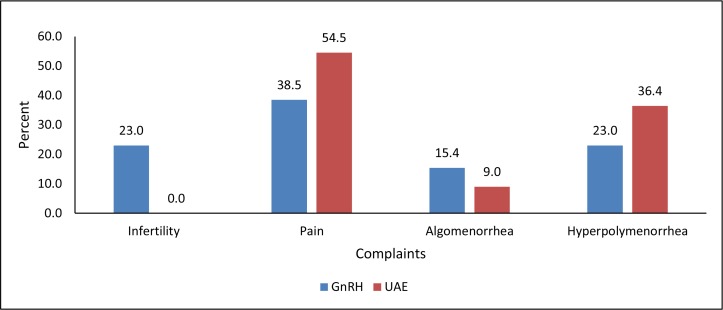
**Breakdown of patients’ complaints stratified by intervention group**

Patients treated with GnRHa (medication group) had a mean age of 34.2±2.2 years, and patients undergoing pre-operative UAE (embolization group) had a mean age of 38.6±1.2 years. Twenty-three percent of the patients in the medication group complained of infertility during the last 4–5 years, where infertility was defined as the inability to conceive after at least one year of unprotected, regular sexual intercourse. Pelvic pain was reported in 38.5% of patients in the medication group, and in 54.5% of patients in the embolization group (*p*<0.05) Algomenorrhea was reported in 15.4% of patients in the medication group, and in 9.0% of patients in the embolization group. Hyperpolymenorrhea was reported in 23% of patients in the medication group and 36.4% of patients the embolization group (*p*<0.05) (Figure 1).

The most common complaints reported by patients in the embolization group included pelvic pain (54.5%) and hyperpolymenorrhea (i.e. dysfunctional uterine bleeding – 36.4%), possibly associated with larger fibroids than observed in the medication group.

**Figure 2.**
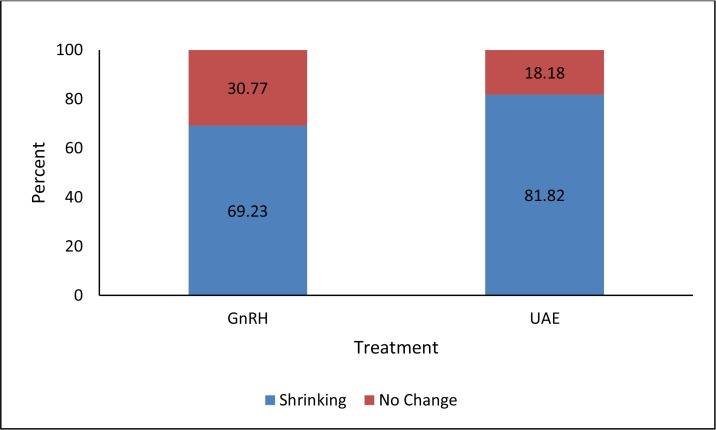
**Reduction of fibroid size after pre-treatment with GnRHa or UAE**

Uterine fibroids ranged in size from 3 to 10 cm in diameter and were located in the sub-serous and intramural layers of the uterus. Based on transvaginal ultrasound examinations, the medication group had single fibroid nodes located in the fundus of the uterus in 38.5% of patients, in the anterior wall in 38.5% of patients, and in combination of the anterior wall with the transition to the fundus (fundal anterior fibroids) overlapping fallopian tubes in 23.0% of patients. The size of fundal fibroids averaged 5.1×4.3×2.0 cm, anterior wall fibroids averaged 5.1×5.0×2.3 cm, and fundal anterior fibroids averaged 6.0×5.8×3.0 cm. In the embolization group, the single fibroid nodes were located at the fundus of the uterus in 18.2% of patients, in the anterior wall in 36.4% of patients, and in the fundal anterior location in 45.5% of patients. The size of the fundal fibroids averaged 6.5×4.2×3.5 cm, the anterior fibroid nodes averaged 10.0×4.0×3.2 cm, and the fundal anterior fibroids averaged 5.7×5.4×3.5 cm. Transvaginal ultrasound demonstrated that 69.2% of patients treated with GnRHa had fibroid shrinkage with the uterine volume decreasing by 25%. The group undergoing UAE treatment had fibroid shrinkage up to 32% among 81.9% of patients (Figure 2). Both treatment groups were comparable in terms of gynecologic and obstetric history.

Surgical times in the medication group were roughly 40 minutes longer than in the embolization group. This is due to the peri-operative difficulties in detecting the layer between myometrium and fibroid capsule. Furthermore, blood loss was much higher in the medication group (130.0±20 ml) compared to the embolization group (80.0±10.0 ml). All patients were encouraged to resume physical activity 8 hours postoperatively, and sutures were removed 3 days after the surgery. Patients were discharged from the hospital 4.0±1.0 days post-surgery. Histological results also confirmed uterine fibroid diagnosis in both groups. Furthermore, both groups had transvaginal uterine ultrasound examinations as follow-up assessments between 6 and 12 months. However, during the 12 month post-surgical follow-up, three patients in the medication group reported uterine fibroid recurrence, whereas the embolization group reported only one case of fibroid recurrence. Pregnancy during the 12 months after surgery was reported in 53.8% of patients in the medication group (*p*<0.01), where 28.6% of women became pregnant at 6 months and 72.4% of women at 9 months after onset of normal menstrual function. Only one patient in the embolization group became pregnant 12 months after recovery, and which previously had anatomical localization of the fibroid nodes at the fundus of the uterus and fundal anterior wall. Overall, this group did not complain of infertility as the primary reason for the decision to undergo embolization. The embolization group only had 2 patients who did not experience fibroid reduction.

## Discussion

This study concluded that despite the fact that both treatments (GnRHa and UAE) were effective for fibroid reduction, embolization resulted in more optimal surgical time, and improved patient outcomes. This is one of the first studies of this nature conducted in Central Asia, as previously published studies from the Asian continent primarily focused on Chinese^8^,^9^ and Japanese^10^,^11^ women.

Limitations of this study included a very small sample size and the retrospective nature of this investigation. An additional limitation of this study is the varying time period between pretreatment with medication or embolization and fibroid surgery. Several women wanted to have surgery at 6 months after pre-treatment, while others waited up to 12 months. Also, this study did not allow for a more detailed evaluation of infertility, since the authors did not have access to women’s reproductive hormone test results and their sexual partners’ spermiogram results.

To date, there is a paucity of published evidence to confirm the safety of uterine artery embolization for the future of reproductive function and fertility. Therefore, we discourage UAE for patients planning to have children. UAE is an acceptable method for surgical preparation, leading to fibroid shrinkage, lower intraoperative blood loss, shorter anesthesia, and surgical times. Based on our experience, the laparoscopic myomectomy after UAE has been advantageous in the identification of fibroid nodules and normal myometrium by fibroid color, consistency, and its borders. Thus, future studies are needed to confirm these findings. Overall, we would like to recommend implementation of a randomized clinical trial for comparison of uterine artery embolization and gonadotropin-releasing hormone agonists in shrinking fibroid size and improving surgical outcomes, specifically in the Central Asian region.

## References

[1] Mahmoud MS, Desai K, Nezhat FR (2015). Leiomyomas beyond the uterus; benign metastasizing leiomyomatosis with paraaortic metastasizing endometriosis and intravenous leiomyomatosis: a case series and review of the literature. Arch Gynecol Obstet.

[2] Szamatowicz M, Kotarski J (2013). [Selective progesterone receptor modulator (ulipristal acetate--a new option in the pharmacological treatment of uterine fibroids in women]. Ginekol Pol.

[3] Gupta JK, Sinha A, Lumsden MA, Hickey M (2014). Uterine artery embolization for symptomatic uterine fibroids. Cochrane database of systematic reviews (Online).

[4] Kamath MS, Kalampokas EE, Kalampokas TE (2014). Use of GnRH analogues pre-operatively for hysteroscopic resection of submucous fibroids: a systematic review and meta-analysis. Eur J Obstet Gynecol Reprod Biol.

[5] Sankaran S, Manyonda IT (2008). Medical management of fibroids. Best Pract Res Clin Obstet Gynaecol.

[6] Nasser F, Affonso BB, de Jesus-Silva SG (2010). [Uterine fibroid embolization in women with giant fibroids]. Revista brasileira de ginecologia e obstetricia : revista da Federacao Brasileira das Sociedades de Ginecologia e Obstetricia.

[7] Bernardo A, Gomes MT, Castro RA, Girao MJ, Bonduki CE, Yokoyama CA (2011). [Impact of the myoma arterial embolization by uterine volume, diameter myoma greater and in the ovarian function]. Revista brasileira de ginecologia e obstetricia : revista da Federacao Brasileira das Sociedades de Ginecologia e Obstetricia.

[8] Zhang Y, Sun L, Guo Y (2014). The impact of preoperative gonadotropin-releasing hormone agonist treatment on women with uterine fibroids: a meta-analysis. Obstet Gynecol Surv.

[9] Yu YH, Gong SP, Wan SM (2004). [Clinical application of GnRHa before uterine myomectomy: report of 20 cases]. Di 1 jun yi da xue xue bao = Academic journal of the first medical college of PLA.

[10] Higashijima T, Kataoka A, Nishida T, Yakushiji M (1996). Gonadotropin-releasing hormone agonist therapy induces apoptosis in uterine leiomyoma. Eur J Obstet Gynecol Reprod Biol.

[11] Uemura T, Mori J, Yoshimura Y, Minaguchi H (1991). Treatment effects of GnRH agonist on the binding of estrogen and progesterone, and the histological findings of uterine leiomyomas. Asia-Oceania journal of obstetrics and gynaecology/AOFOG.

